# Photodynamic Activity of Chlorophyllin and Polyethylenimine on *Pseudomonas aeruginosa* Planktonic, Biofilm and Persister Cells

**DOI:** 10.3390/ijms241512098

**Published:** 2023-07-28

**Authors:** Mona Mahmoud, Peter Richter, Michael Lebert, Andreas Burkovski

**Affiliations:** 1 Department of Biology, Microbiology Division, Friedrich-Alexander-Universität Erlangen-Nürnberg, 91058 Erlangen, Germany; mona.mahmoud@fau.de (M.M.); andreas.burkovski@fau.de (A.B.); 2 Dairy Department, National Research Centre, Dokki, Giza 12622, Egypt; 3 Gravitational Biology Group, Department of Biology, Cell Biology Division, Friedrich-Alexander-Universität Erlangen-Nürnberg, 91058 Erlangen, Germany; michael.lebert@fau.de; 4 Space Biology Unlimited S.A.S., 33000 Bordeaux, France

**Keywords:** antimicrobial resistance, dormant bacteria, photosensitizer, stringent response

## Abstract

Antimicrobial photodynamic inactivation is considered a promising antimicrobial approach that may not develop resistance in the near future. Here, we investigate the influence of the photosensitizer chlorophyllin (CHL) and the cationic permeabilizer polyethylenimine (PEI), exposed to a red light-emitting diode, on the human pathogen *Pseudomonas aeruginosa* free-living planktonic cells, the sessile biofilm and persister cells. The broth microdilution checkerboard method was used to test antimicrobial susceptibility. As a substrate for biofilms, the Calgary biofilm device was used, and the quantification of the biofilm biomass was carried out using a crystal violet assay. Serine hydroxamate was used for the induction of persisters. Our findings reveal that PEI ameliorates the antimicrobial activity of CHL against *P. aeruginosa* planktonic and biofilm states, and the concentration required to eradicate the bacteria in the biofilm is more than fourfold that is required to eradicate planktonic cells. Interestingly, the persister cells are more susceptible to CHL/PEI (31.25/100 µg mL^−1^) than the growing cells by 1.7 ± 0.12 and 0.4 ± 0.1 log_10_ reduction, respectively, after 15 min of illumination. These data demonstrate that CHL excited with red light together with PEI is promising for the eradication of *P. aeruginosa*, and the susceptibility of *P. aeruginosa* to CHL/PEI is influenced by the concentrations and the exposure time.

## 1. Introduction

The emergence of new antimicrobial-resistant pathogens represents a global challenge due to the transfer of bacteria between the environment, humans and animals [1]. During this transfer, pathogens acquire new antibiotic-resistant genetic elements, thus hindering our ability to treat bacterial infections [1]. In 2014, it was estimated that by 2050 the death rate would be 10 million due to resistant infections [2]. Moreover, more than 60% of nosocomial infections are caused by biofilm-producing microbes, which is considered a major clinical challenge due to their extracellular polymeric matrix that is difficult to penetrate [3]. Microbial biofilm can tolerate up to 100–1000 times more antibiotics than their planktonic-free cell counterparts [4]. Therefore, the WHO reported in 2020 that antibiotic resistance is a global challenge and possesses a high priority for promoting research and developing novel antimicrobials against multidrug-resistant pathogens [5]. Unfortunately, antibiotic resistance is still in progress, and there were an estimated 4.95 million deaths associated with drug-resistant infections in 2019, of which 1.27 million deaths were directly attributed to drug resistance [6]. One of the leading pathogens contributing to the burden of antimicrobial resistance in 2019 and the priority target for the development of alternative therapeutics and new antimicrobial agents is the opportunistic pathogen *Pseudomonas aeruginosa* [6,7].

*P. aeruginosa* is a ubiquitous Gram-negative bacterium considered a leading pathogen in immunocompromised patients [8]. This bacterium is dominant in patients with cystic fibrosis because of its ability to promote the establishment of a microbial community in the airways, leading to prolonged chronic infections and rapid lung damage and mortality [9]. Furthermore, it is an important causative agent of chronic wound infections [10]. Biofilm formation, pigment secretion of pyocyanin and protease production make *P. aeruginosa* a major food spoilage bacterium, and its presence in water can contaminate food and can transfer from infected asymptomatic animals to the excreted milk and meat [11,12,13]. The physiological and genetic determinants of *P. aeruginosa* make it resilient to environmental changes and cause persistent infections that are difficult to treat [8]. The low susceptibility of most *P. aeruginosa* strains to many antibiotics arises from their intrinsic core genome, which encodes virulence and antibiotic-resistant determinants [14]. Moreover, their ability to produce a biofilm leads to more antibiotic tolerance and antimicrobial failure. A biofilm is a heterogeneous and dynamic microenvironment; it is a set of different phenotypes, including susceptible actively growing cells, resistant cells and dormant persister cells [15,16]. The diversity of the biofilm population arises as a consequence of the nutrient and oxygen gradient in the biofilm while growing, which changes the phenotypic characteristics and gene expressions of the embedded cells [15,16]. Therefore, the development of new antimicrobial agents against *P. aeruginosa* is urgently needed [8,15].

One of the promising antimicrobial approaches that may overcome the development of resistance in the near future is photodynamic inactivation [17,18]. The principle of photodynamic inactivation (PDI), antimicrobial photodynamic therapy (PDT), or antimicrobial photodynamic inactivation (aPDI) is based on the excitation of a non-toxic photosensitizer (PS) compound with a light source with a spectrum appropriate for the excitation of the PS (from the visible to infrared spectrum) in the presence of molecular oxygen, resulting in the generation of reactive oxygen species targeting different cellular structures and metabolic pathways [17,19]. aPDI may lead to bacterial eradication or irreversible changes in the bacterial cells in such a way that it is difficult for the bacterial cell to recover or form resistance [18]. Therefore, aPDI has a low risk of causing resistance [18]. Porphyrin molecules have great potential against pathogenic bacteria [20] and are promising candidates in wastewater disinfection [21]. A highly promising plant-based porphyrin photosensitizer, which was shown to exhibit antimicrobial activity against Gram-positive (*Bacillus subtilis*) and Gram-negative (*Escherichia coli*, *Salmonella enterica Serovar Typhimurium*) bacteria, is chlorophyllin [22,23,24,25]. Chlorophyllin (CHL) is a semisynthetic, water-soluble, due to the removal of the phytol group, and green food colorant, derived from chlorophyll [19,26]. In addition to the inactivation of microorganisms, chlorophyllin has antimutagenic properties and is capable of restoring the gut microbial balance [26,27].

Up to now, the photodynamic activity of CHL has not been studied with respect to the inactivation of *P. aeruginosa*, and little research has been performed on the excitation of CHL with red light and its efficacy against bacterial biofilm and persister cells. Due to the presence of an outer membrane as part of the cell envelope, the eradication of Gram-negative bacteria such as *P. aeruginosa* is expected to be more difficult to achieve than in the case of Gram positives [28]. It was reported that the penetration of the Gram-negative cell envelope can be enhanced using cationic PS or in combination with positively charged permeabilizing agents, which can increase the permeability of the outer membrane and may enhance the efficiency of the inactivation [28,29].

In this study, we tested the excitation of CHL using red light-emitting diodes (LED), and polyethylenimine (PEI) was used as an adjuvant material aiming at eradicating *P. aeruginosa* free-living planktonic cells, biofilm and persister cells.

## 2. Results

### 2.1. Photostability of Chlorophyllin

**Figure 1:**
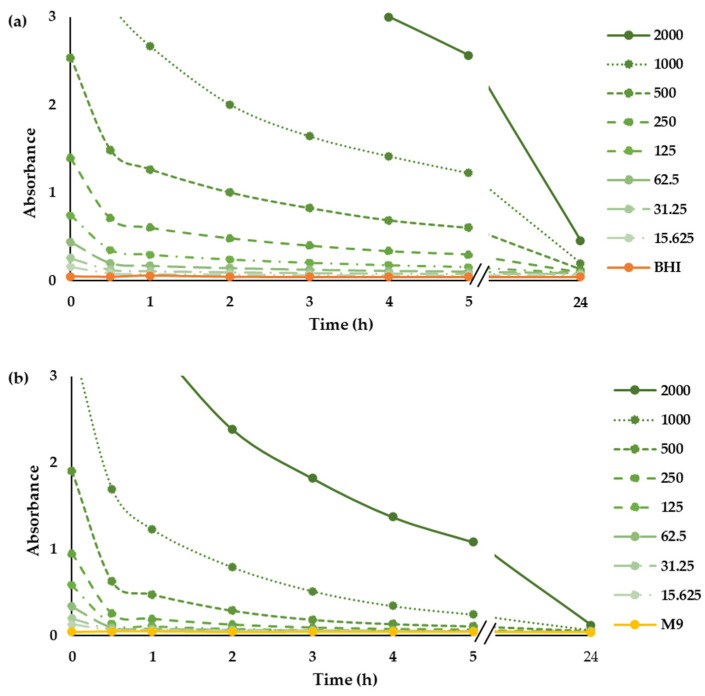
Photostability of CHL. Time-dependent absorption spectrum of CHL (µg mL^−1^) at 660 nm in BHI (**a**) and M9 (**b**) medium.

In order to determine the photostability of CHL in the growth media used in this study, we determined the absorption spectrum of the whole tested concentrations of CHL in BHI and M9 before and after illumination with red LEDs (Figure S1). Before illumination, CHL had an absorption band with a maximum peak at 660 nm, and the absorbance increased with increasing the concentration. Depending on illumination time, the absorbance is reduced, and the visible peaks diminish (Figure S1), indicating the degradation of CHL (Figure 1). The degradation of CHL in the M9 minimal medium was approximately 40% faster than in BHI complex medium. Similarly, Nie and colleagues [30] observed a reduction in the absorbance of the chlorophyll derivative Chlorine e6 after illumination with red LEDs, demonstrating its degradation.

### 2.2. Influence of Polyethylenimine on the Eradication of P. aeruginosa Planktonic Cells by Chlorophyllin

**Figure 2:**
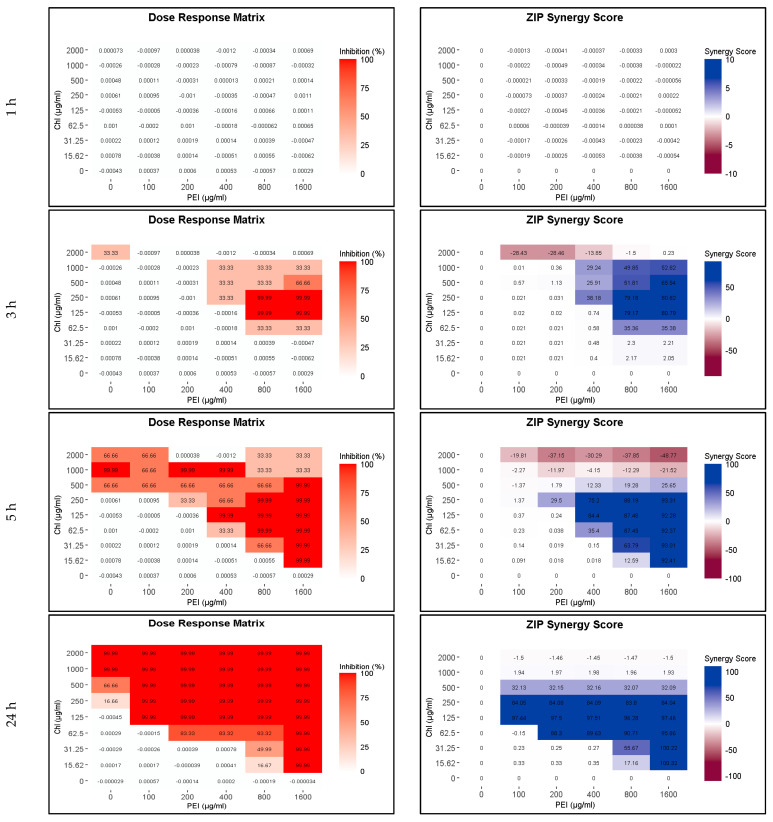
Influence of illuminated chlorophyllin (CHL) and polyethylenimine (PEI) on *P. aeruginosa* planktonic cells after exposure to the red light for 1, 3, 5 and 24 h. The dose–response matrices show the eradication percentage (4 log_10_ reduction) of the double serial dilution of CHL and PEI individually and in combination. ZIP (zero interaction potency) synergy score matrices depict the effect of PEI. The matrices were plotted using RStudio SynergyFinder R package. The results are an average of three biological replicates (exposure time 1–5 h) and six biological replicates (exposure time 24 h).

PEI potentiates the action of β-lactam antibiotics against methicillin-resistant *Staphylococcus aureus* [31], and recently, we found that PEI potentiates the efficacy of CHL against *E. coli* [25]. In the first step, we examined whether PEI could also potentiate the function of CHL against P. aeruginosa. Between 0 and 2000 µg mL^−1^ of CHL and between 0 and 1600 µg mL^−1^ of PEI were tested alone and in combination (Figure 2). Unilluminated CHL, PEI alone and the red-light source had no lethal effect under the tested conditions. Compared to the individual compounds, which have no bactericidal effect after 3 h of incubation, the combination of 125 µg mL^−1^ CHL and 800 µg mL^−1^ PEI led to the eradication of bacteria by 99.99% (4 log_10_ reduction). By increasing the exposure time to 5 h, the minimal bactericidal concentration (MBC) was reduced twofold to 125 µg mL^−1^ CHL and 400 µg mL^−1^ PEI (Figure 2). After 24 h, the highest tested concentrations of the illuminated CHL alone (1000 and 2000 µg mL^−1^) eradicated the planktonic cells by 99.99% (Figure 2). The concentration of 100 µg mL^−1^ PEI potentiated a lower concentration of CHL (125 µg mL^−1^) to exert the same effect, with a potentiation score of 97% shown in the dose–response and the synergy score matrices in Figure 2. The staircase pattern in the dose–response matrices indicates the efficacy of multiple combinations against planktonic cells. These results prove that the lethal effect of lower doses of the CHL and PEI increases with increasing exposure time, and the effect of the CHL alone started to appear after 5 h.

### 2.3. Effect of Chlorophyllin and Polyethylenimine on Sessile P. aeruginosa

#### 2.3.1. Influence on Biofilm

**Figure 3:**
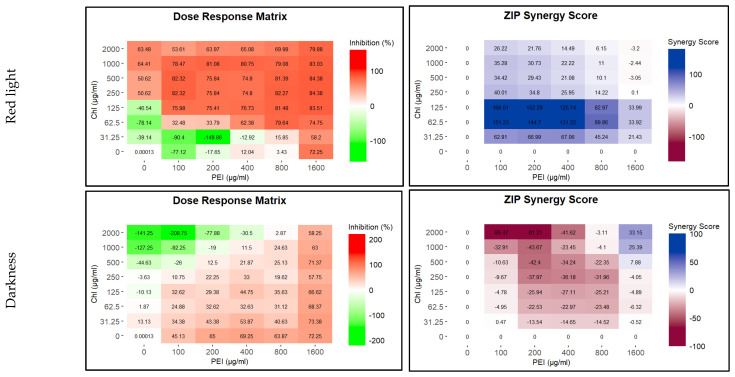
Influence of illuminated and unilluminated chlorophyllin (CHL) and polyethylenimine (PEI) on *P. aeruginosa* biofilm. The dose–response matrices show the detachment percentage of the double serial dilution of CHL and PEI individually and in combination. ZIP (zero interaction potency) synergy score matrices depict the synergistic and antagonistic effect between CHL and PEI. The matrices were plotted using RStudio SynergyFinder R package. The results are an average of eight biological replicates.

To test the efficacy of the CHL and PEI on the established biofilm, we used the Calgary biofilm device for biofilm formation and crystal violet for biofilm biomass quantification. BHI was the best medium for biofilm formation on the polystyrene peg lid. As shown in Figure S2, there were no significant differences between the tested incubation times. Similar to the effect of sub-lethal doses of antibiotics on biofilm formation [32,33], lower concentrations of illuminated CHL and PEI stimulated biofilm formation, while higher concentrations dislodged the biofilm. Synergistically, illuminated CHL (125 µg mL^−1^) and PEI (100 µg mL^−1^) dislodged the established biofilm by approximately 76% with a synergy score exceeding 100%. In contrast, higher concentrations of the unilluminated CHL stimulated biofilm formation (Figure 3).

#### 2.3.2. Eradication of *P. aeruginosa* Cells in Biofilm

**Figure 4:**
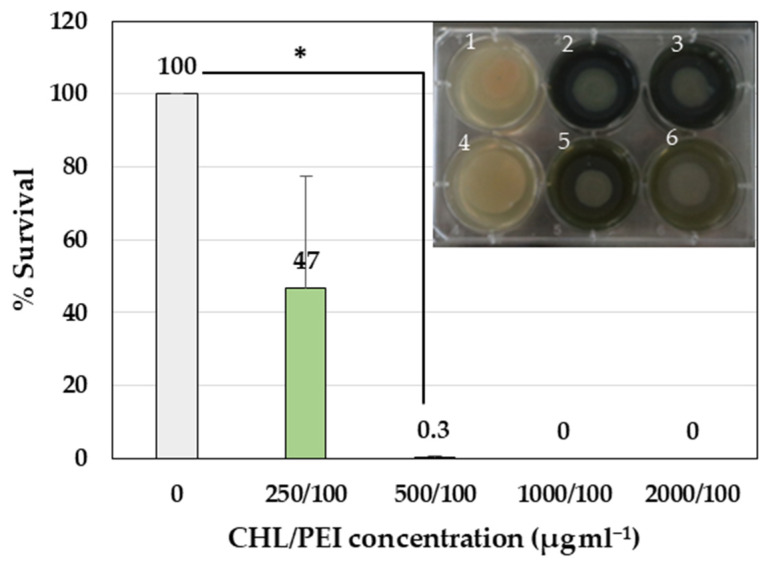
Antibiofilm activity of CHL and PEI on established colony biofilm. Error bars represent standard deviation of the mean (*n* = 3). The photo represents the established *P. aeruginosa* colony biofilm on a polycarbonate membrane on BHI agar (1: growth control, 2: CHL/PEI 2000/100 µg mL^−1^, 3: CHL/PEI 1000/100 µg mL^−1^, 4: sterility control, 5: CHL/PEI 500/100 µg mL^−1^, 6: CHL/PEI 250/100 µg mL^−1^). The untreated sample was normalized at 100%. * *t*-test: *p*-value < 0.05.

To test the effect of CHL and PEI on the viability of the biofilm-embedded bacteria, we tested the effect of the four concentrations of CHL (2000, 1000, 500 and 250 µg mL^−1^) in combination with 100 µg mL^−1^ PEI on established colony biofilms. Treatment with concentrations of 500 µg mL^−1^ CHL in the presence of 100 µg mL^−1^ PEI had a significant (*p* < 0.05) eradication effect (99.7%) against the bacteria in the biofilm compared to the control without treatment (Figure 4).

### 2.4. Analysis of Persister Cells

#### 2.4.1. Induction of Persister Cells by the Stringent Response

**Figure 5:**
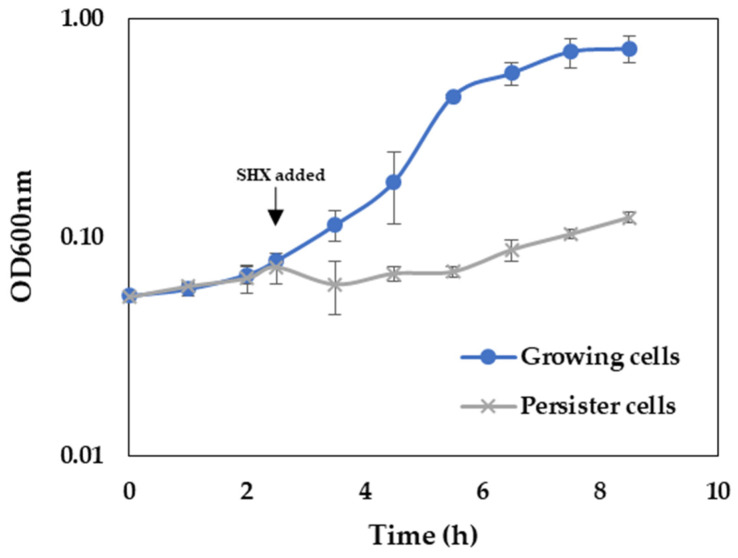
Influence of SHX addition on growth of *P. aeruginosa*. Bacteria were grown in M9 minimal medium without and with the addition of 500 µg mL^−1^ SHX.

The persistence of *P. aeruginosa* can be induced by amino acid starvation and subsequent induction of stringent response [34,35]. For this purpose, the amino acid analog serine hydroxamate (SHX) was added to the M9 medium, and growth was monitored (Figure 5). As expected, in response to SHX addition, a growth arrest of *P. aeruginosa* was observed.

**Figure 6:**
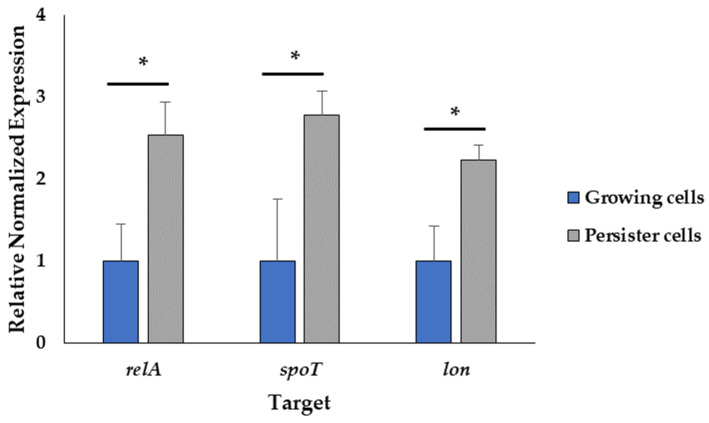
Induction of the stringent response. The normalized gene expression relative to the reference gene *rpoS* of the treated and untreated samples using the 2^−ΔΔCt^ method. Data represent the mean value of three biological replicates with three technical replicates (*n* = 9). Error bars represent the standard deviations. * *t*-test: *p*-value < 0.05.

To further confirm the induction of the stringent response from the addition of SHX, we measured the transcript levels of the *relA* and *spoT* genes, which encode the key proteins of the stringent response regulating the (p)ppGpp alarmone levels in response to starvation [35]. In addition, the transcript level of the *lon* (encoding lon protease) gene was tested since the corresponding protein is involved in arresting growth in favor of bacterial survival under starvation conditions [35]. The relative expression of the target genes was normalized to *rpoS* as a reference gene. The stability of *rpoS* under the experimental conditions was assessed based on Cohen’s d [36,37], showing a small effect size of 0.2, as illustrated in Figure S3. The transcript levels of *relA*, *spot* and *lon* increased approximately 2.53, 2.77 and 2.23-fold, respectively, upon SHX addition (Figure 6). This result corroborates that the experimental condition induced persister cells.

#### 2.4.2. Effect of Chlorophyllin and Polyethylenimine on *P. aeruginosa* Persister Cells

**Figure 7:**
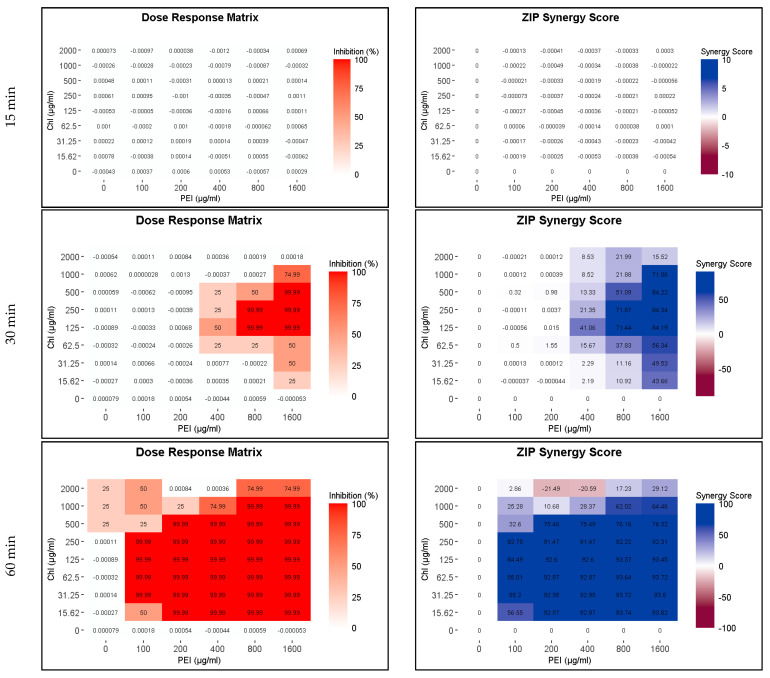
Influence of illuminated chlorophyllin (CHL) and polyethylenimine (PEI) on *P. aeruginosa* growing cells. The dose–response matrices show the effect of the double serial dilution of CHL and PEI individually and in combination. ZIP (zero interaction potency) synergy score matrices depict the synergistic effect between CHL and PEI. The matrices were plotted using RStudio SynergyFinder R package. The results are an average of four biological replicates.

**Figure 8:**
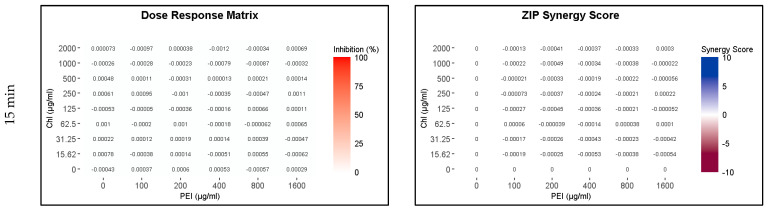

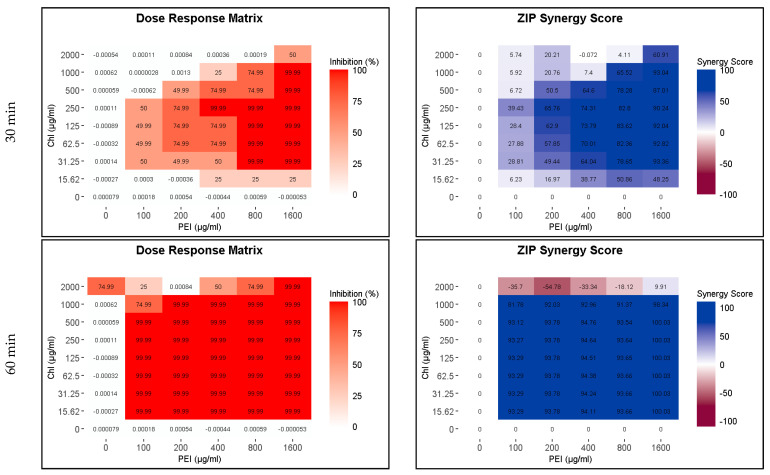
Influence of illuminated chlorophyllin (CHL) and polyethylenimine (PEI) on *P. aeruginosa* persister cells. The dose–response matrices show the effect of the double serial dilution of CHL and PEI individually and in combination. ZIP (zero interaction potency) synergy score matrices depict the synergistic effect between CHL and PEI. The matrices were plotted using RStudio SynergyFinder R package. The results are an average of four biological replicates.

Because of the higher susceptibility of the persister and growing cells to CHL and PEI in the M9 minimal medium compared to that in the BHI medium and the faster rate of degradation of CHL in M9 compared to BHI (Figure 2 and Figure S1), the exposure time was reduced to 15, 30 and 60 min. The lethal effect (4 log_10_ reduction) of the tested combination appeared after 30 min of exposure to red light and lower concentrations exerted the same effect by doubling the exposure time (Figure 7 and Figure 8). Unilluminated CHL, PEI alone and the red-light source had no lethal effect under the tested conditions, making the synergistic effect of the tested combinations more obvious (Figure 7 and Figure 8). The dose–response matrices in Figure 7 showed that 800 µg mL^−1^ PEI and 125 µg mL^−1^ CHL were required to eradicate the growing cells after 30 min of exposure, whereas half of the concentration of the PEI (400 µg mL^−1^) and double the concentration of the CHL (250 µg mL^−1^) were required to exert the same effect on the persister cells (Figure 8). After 60 min of exposure, the eradication rate increased with the lower tested concentrations of CHL/PEI 31.25/100 µg mL^−1^ for the growing cells and CHL/PEI 15.62/100 µg mL^−1^ for the persister cells (Figure 7 and Figure 8). This result suggests that persister cells are more vulnerable to CHL and PEI than the growing cells, and CHL alone requires more exposure time to exert its antimicrobial action.

**Figure 9:**
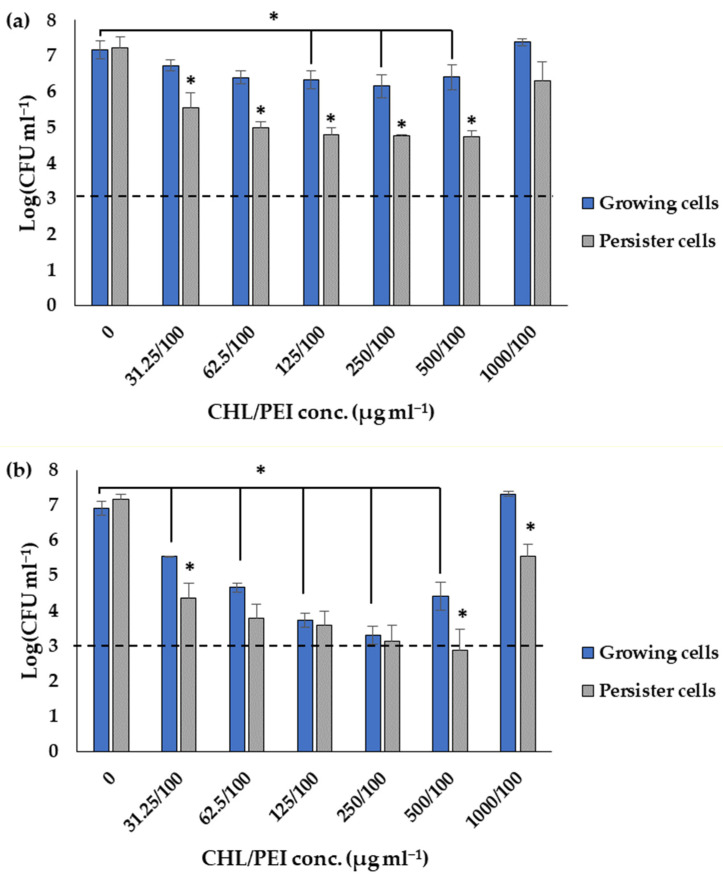
Influence of different concentrations of chlorophyllin (CHL) and 100 µg mL^−1^ polyethylenimine (PEI) on growing and persister cells after exposure to red LEDs for 15 min (**a**) and 30 min (**b**). The dashed line corresponds to a 4 log_10_ reduction (99.99% killing efficacy). Data represent the mean value of three biological replicates. Error bars represent the standard deviations. * *t*-test: *p*-value < 0.05.

In order to prove the result of the dose–response matrices, the efficacy of 100 µg mL^−1^ PEI and different concentrations of CHL were tested via viable cell count. The results in Figure 9 corroborated that the persister cells are more sensitive than the growing cells to the lowest tested concentrations of 100 µg mL^−1^ PEI and 31.25 µg mL^−1^ CHL with 1.3 and 1.4 log_10_ reductions after 15 and 30 min, respectively. The growing cells were reduced by 0.4 ± 0.1 log_10_ after 15 min of exposure and significantly (*p* < 0.05) reduced by 1.4 ± 0.17 log_10_ after 30 min exposure at CHL/PEI 31.25/100 µg mL^−1^, whereas the persister cells were significantly (*p* < 0.05) reduced by 1.7 ± 0.12 and 2.8 ± 0.31 log_10_, respectively, under the same conditions. It is also noted that the CHL at concentration 1000 µg mL^−1^ in presence of PEI 100 µg mL^−1^ is not lethal for the growing cells while significantly (*p* < 0.05) reducing the persister cells by 1.6 log_10_ after 30 min of exposure to aPDI, indicating a non-linear response. These data, in conjunction with the previous investigations mentioned above, demonstrate that the response of *P. aeruginosa* to CHL and PEI is governed by concentration-dependent and time-dependent mechanisms.

## 3. Discussion

The global burden of antibiotic resistance continues to grow, and the development of new antibacterial agents has been inadequate in tackling this growing threat. In particular, *P. aeruginosa* is not only a leading opportunistic human pathogen but also a prevalent food spoilage bacterium in various food products, especially in those with higher water content [38]. Herein, we utilize aPDI via the excitation of CHL with red light (λ 623 nm, 295.55 J/m^2^/s) individually and together with PEI against *P. aeruginosa* planktonic cells, biofilm and persister cells. This study demonstrates that PEI enhanced the efficiency of CHL in the eradication of the planktonic free cells and biofilm cells, and more than fourfold of the antimicrobial concentrations (CHL/PEI > 500/100 µg mL^−1^) are required to eradicate the bacteria in biofilm (>99.99%, >4 log_10_ reduction) compared to planktonic cells (CHL/PEI 125/100 µg mL^−1^). In addition, the CHL/PEI 125/100 µg mL^−1^ combination is capable of detaching the established biofilm by approximately 76%. Surprisingly, the CHL/PEI (31.25/100 µg mL^−1^) combination was more effective against persister cells compared to the growing cells by 1.7 ± 0.12 and 0.4 ± 0.1 log_10_ reduction, respectively, after 15 min of exposure to red LEDs, suggesting that the combinations can overcome the tolerance caused by the dormant cells.

CHL is a negatively charged PS and has electronic absorption at longer wavelengths (660 nm), as shown in Figure S1. Thus, the long absorption wavelength allows CHL to be a potential candidate for deep penetration [28]. Under the light irradiation of PS with an appropriate wavelength, the PS can undergo an electron transfer reaction (Type I mechanism) with triplet (ground-state) oxygen (^3^O_2_) to produce reactive oxygen species (ROS) such as superoxide radical anion O_2_^−•^, hydrogen peroxide H_2_O_2_, hydroxyl radical HO^•^ or energy transfer reactions (Type II mechanism) to activate (ground-state) oxygen to form singlet (excited-state) oxygen (^1^O_2_) [39]. Singlet (excited-state) oxygen has a short half-time of about 200 ns in cells [40], ranging from 100 ns in the lipid areas of membranes to 250 ns in the cytoplasm [41]. The possible diffusion distance of ^1^O_2_ in a biological system could be up to 10 nm [40]. Therefore, it reacts with the immediate neighborhood target molecules [40]. The chlorophyll triplet state has an even longer lifetime (a few µs) and under O_2_-saturated conditions, can react with ^3^O_2_ to produce the very reactive ^1^O_2_ if no efficient quenchers are around [40]. Wang and colleagues [42] found that the irradiation of CHL with visible light for 30 min generated ROS including ^1^O_2_ and HO^•^. Furthermore, Buchovec and colleagues [24] found that a tiny part of CHL bound to *Salmonella enterica* serovar Typhimurium and the presence of an ^1^O_2_ scavenger (sodium azide NaN_3_) protected the cells from the phototoxic effect of CHL. It is well documented that the outer membrane of the Gram-negative bacteria acts as a physical barrier against PSs reaching the cell membrane. Hence, positively charged PSs generally lead to the more effective inactivation of Gram-negative bacteria than negatively charged and neutral PSs because of their ability to bind to the negatively-charged phosphate groups of the outer membrane and, when excited, contribute to damage to membrane-building lipids and proteins, including membrane-associated enzymes [28]. In our study, the MBC of CHL alone was 1000 µg mL^−1^ with a long exposure time of 5 h against *P. aeruginosa* (Figure 2). Taken together with the above-mentioned previous investigations, the possible explanation of the efficacy of CHL alone could be that the generation of the highly reactive ^1^O_2_ and other ROS that are capable of oxidizing proteins, lipids and nucleic acids requires a high concentration of CHL and a long illumination time and could occur without the necessity for direct contact between CHL and the cells. Moreover, the obtained results proved that the unilluminated CHL and the red light itself have no bactericidal effect on the *P. aeruginosa* cells at the tested conditions, suggesting that *P. aeruginosa* do not have to engage in adaptive survival mechanisms against CHL [19]. This result is consistent with López-Carballo and colleagues [43], who studied the susceptibility of *Staphylococcus aureus*, *Listeria monocytogenes*, *E. coli* and *Salmonella* spp. to CHL incorporated into gelatin film with no toxicity of the dark controls.

The branched 25 kDa PEI Is a positively charged aliphatic polymer that contains amino nitrogen at every third atom of the polymer backbone as a mixture of primary, secondary, and tertiary amines, which provides its cationic nature upon protonation [44,45]. This cationic characteristic can elicit apoptosis in eukaryotic cells [46]. However, branched 25 kDa PEI is widely used as a transfection agent and has various medical applications because it is a good compromise between toxicity and efficacy [47,48]. We speculate that the electrostatic interaction between CHL and PEI or the ROS generated during the photodynamic process could open the door and be an advantage for the reduction in the cytotoxicity of PEI via the reduction in the cationic charges over the surface of the molecule, as it was previously proven that PEI/DNA complexes were less toxic by about 10% than PEI alone [49]. Moreover, oxidizing the amine groups of the branched 25 kDa PEI by hydrogen peroxide reduced its surface charge and its cytotoxicity [44]. Indeed, further investigations are needed to determine the cytotoxicity of the effective combinations of our study for a deeper understanding of the interaction between CHL and PEI. In the whole tested conditions in our study, PEI 25 kDa alone had no direct lethal effect on *P. aeruginosa*. Similarly, Helander et al. [50] found that PEI 50 kDa enhanced the susceptibility of the Gram-negative bacteria *E. coli*, *P. aeruginosa* and *Salmonella* spp. to antibiotics without being directly lethal. On the other hand, 64 µg/mL of the branched PEI 1800 Da reduced the viability of *E. coli* by 60% after 2 h, while the cell reduction was approximately 97% of the Gram-positive bacteria *Staphylococcus aureus* in 10 min, suggesting that the cell structure played a role in the antibacterial activity of PEI [51].

*P. aeruginosa* is recognized as a paradigm bacterium for biofilm formation because of its ability to adhere to a variety of surfaces, including human tissues, instruments and food; therefore, dislodging its biofilm is necessary [38]. Our study demonstrated that PEI (1600 µg mL^−1^) can detach approximately 72% of the established biofilm, and only 100 µg mL^−1^ PEI in the presence of CHL at 125 µg mL^−1^ under illumination conditions was required to detach approximately 76% of the biofilm. However, more than CHL/PEI 500/100 µg mL^−1^ was required to eradicate the bacteria in the colony biofilm. Similarly, Panlilio and colleagues [52] proved that *P. aeruginosa* biofilm can be disrupted via PEI linked with polyethylene glycol. The cationic nature of the PEI most probably destabilizes the biofilm components such as the eDNA, proteins and extracellular polymeric matrix via electrostatic interaction [52]. Therefore, PEI could help the deeper penetration of the CHL in order to exert its action against the biofilm life forms.

It is well known that the persister cells are less susceptible to antimicrobial agents than the growing cells and their predominant role in treatment failure [53,54]. Interestingly, our study revealed that the persister cells are more vulnerable to CHL and PEI combinations than the growing cells. CHL/PEI 31.25/100 µg mL^−1^ eradicated the persister cells by a 1.7 ± 0.12 log_10_ reduction after 15 min of exposure to red LED, whereas the same condition eradicated the growing cells by a 0.4 ± 0.1 log_10_ reduction. This could be because of the reduced membrane potential of the persister cells [55] and the presence of the cationic polymer PEI, which facilitates the phototoxicity of CHL besides the ROS that may not require active target sites to exert their effects. In the study by Roy and colleagues [55], minocycline antibiotic was more effective against *E. coli* persister cells than growing cells. The authors proved that the reduced membrane potential and drug efflux of the persister cells facilitated the penetration and accumulation of the antibiotic inside the cells and killed the cells while reverting to metabolically active cells.

Based on the above mentioned, the results emphasized the synergism between CHL and PEI and the possibility of eradicating persister cells via aPDI. However, more research is required to determine the mechanism behind this, which is a part of our ongoing research for better insights into the potential applications. Chlorophyllin could be a promising candidate in various applications, for instance, wound healing, food preservation and water purification. However, to fully understand its benefits, limitations and safety in each specific context, further research and rigorous testing, including the optimization of the light doses, are necessary.

## 4. Materials and Methods

### 4.1. Chemicals

**Figure 10:**
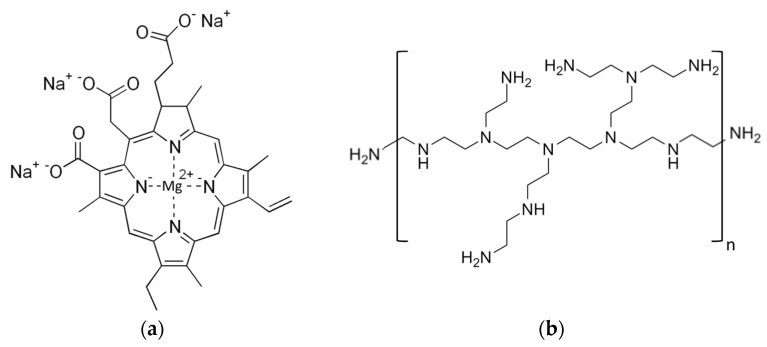
Chemical structure of chlorophyllin sodium salt (**a**) and polyethylenimine (**b**).

Non-copperized chlorophyllin sodium salt (CHL), Mw 684.9 g mol^−1^, CAS number 15203-43-7, was purchased from Carl Roth GmbH (Karlsruhe, Germany). Polyethylenimine (PEI), 25 kDa branched, CAS number 9002-98-6, was purchased from Sigma-Aldrich (St. Louis, MO, USA). For chemical structures, see Figure 10.

### 4.2. LED-Based Light Source

**Figure 11:**
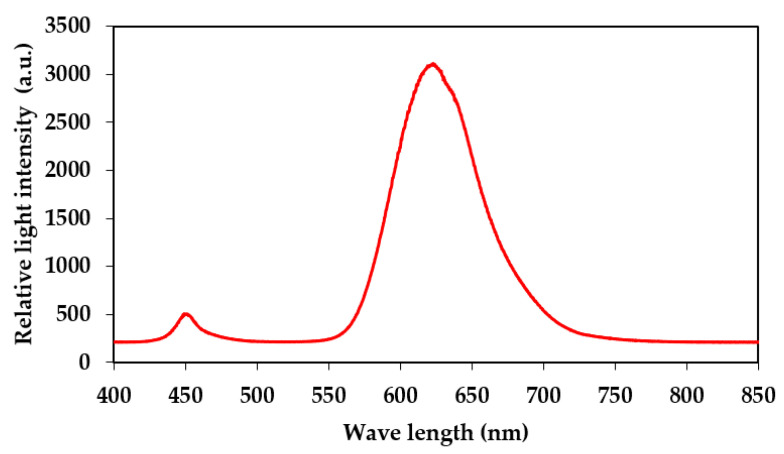
Emission spectrum of applied red LED-based light source.

A red LED light source for aPDI was obtained from Shenzhen Cooleeon Electronics Co., Ltd., Shenzen, China. The LED emission spectrum was tested using an Ocean Optics usb2000 spectrophotometer with the average maximum emission spectrum of the LEDs being 623 nm (Figure 11). Samples in 6-well or 96-well plates were placed 10 cm away from the LED source to ensure the equal distribution of the light, and a fan was placed near the construction to avoid temperature increase during the irradiation process. The light intensity measured at the surface of the plates using quantum radiation probe FLA 623 PS connected to an ALMEMO 2490 device (Ahlborn, Mess- und Regelungtechnik GmbH, Holzkirchen, Germany) was 1537 ± 130 µmol m^−2^ s^−1^. This light intensity corresponds to 295.55 J m^−2^ s^−1^.

The absorption spectrum of CHL solution was recorded using an absorption scan between 400 and 800 nm in 96-well flat bottom cell culture plates (Greiner Bio-One, catalog no. 655180, Frickenhausen, Germany) using a plate reader (infinite 200Pro, Tecan, Männedorf, Switzerland).

### 4.3. Growth Conditions and Photodynamic Inactivation

*P. aeruginosa* strain DSM 50071 (DSMZ, Braunschweig, Germany) was used as a Type strain. The bacteria were routinely grown in Brain Heart Infusion (BHI; Oxoid, Wesel, Germany) at 37 °C. For each experiment, the bacteria were plated out from the 30% glycerol frozen stocks onto BHI agar plates and incubated at 37 °C for 24 h. A single colony was picked from the agar plate, inoculated in BHI broth and incubated at 37 °C for 16 to 17 h with agitation at 125 rpm for preparation of overnight culture.

**Figure 12:**
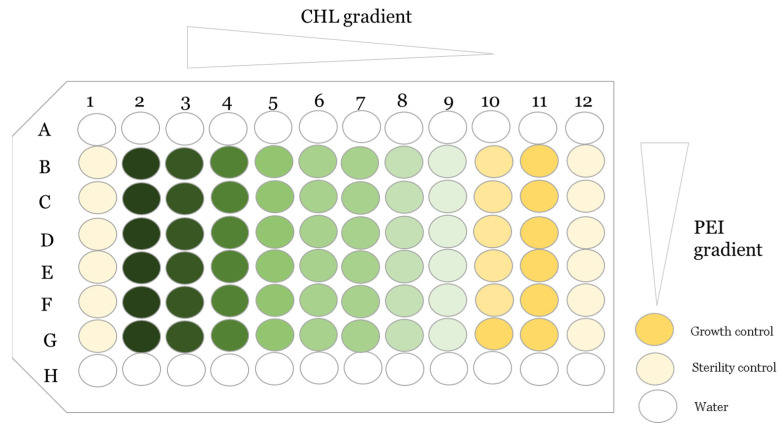
An overview of the antimicrobial susceptibility testing using broth microdilution checkerboard assay (the challenge plate). The green color refers to the chlorophyllin (CHL) gradient alone in row G, and the light yellow color in column 10 refers to the PEI gradient alone. The rest are gradient of CHL/PEI.

In order to evaluate the bactericidal and the biofilm disruption of CHL and PEI alone and in combination, antimicrobial susceptibility testing was performed using broth microdilution checkerboard assay [56]. As described in Figure 12, the peripheral wells were filled with sterile distilled water to reduce dehydration throughout the incubation time. Negative control without the bacteria, checking the non-contamination, and positive growth control without the antimicrobial compounds were added, followed by twofold dilutions of each antimicrobial in the inner 60 wells. To test the susceptibility of the planktonic cells to the tested antimicrobials, a diluted overnight culture adjusted to an optical density at 600 nm (OD_600_) of 0.1 corresponding to 4 ± 1 × 10^7^ colony-forming units (CFU) mL^−1^ was used to inoculate the microtiter plate (100 µL per well), then covered with transparent seal (Greiner, Frickenhausen, Germany) to avoid cross-contamination, followed by exposure to red light. In order to determine the minimum bactericidal concentration (MBC), aliquots (3 µL) from each well were subcultured on BHI agar. A dark control was evaluated in parallel.

### 4.4. Biofilm Formation and Quantification

As a substrate for biofilm, the Calgary biofilm device was used [57]. Briefly, overnight culture diluted to OD_600_ = 0.1 in fresh BHI was used to inoculate a 96-well microtiter plate (Thermo Fisher scientific, Roskilide, Denmark) using 150 µL per well; the outer wells were used for sterility controls and water. The plate was then covered with the peg lid (96 polystyrene pegs, Thermo Fisher scientific, Roskilide, Denmark) and incubated at 37 °C for 24 h under constant agitation. After 24 h, the biofilm was rinsed in PBS for 1 min and subjected to a new plate containing CHL and PEI in BHI for 24 h. Quantification of the biofilm biomass was carried out using crystal violet [58]. Briefly, the peg lid with the biofilm was rinsed in PBS for 1 min and incubated at 65 °C for 2 h for drying, followed by immersion in crystal violet (Neisser-Solution II, Carl Roth, Karlsruhe, Germany) for 15 min. Subsequently, the stained pegs were rinsed in distilled water and immersed in 33% acetic acid, and absorbance was measured using a plate reader at 570 nm. The percentage of the biofilm disruption was calculated via normalization of the untreated samples.

### 4.5. Characterization of Bacterial Viability in Established Colony Biofilms

In order to determine the efficacy of the tested CHL and PEI combinations on bacteria embedded in biofilm matrix, *P. aeruginosa* was grown as a colony biofilm as previously described [34] with some modifications. Briefly, 10 µL from a diluted overnight culture (see above) were transferred to a sterilized polycarbonate membrane filter (25 mm diameter, 0.8 µm pore size, Whatman, Shrewsbury, MA, USA) placed on BHI agar plate. Plates were incubated at 37 °C for 4 days. Filters with the biofilm were subsequently transferred to a 6-well plate: a well for the sterility control, the membrane without the biofilm, a well for the positive control, membrane with biofilm on BHI agar without any antimicrobials and the rest four wells containing BHI agar mixed with different concentrations of CHL (2000, 1000, 500 and 250 µg mL^−1^) and fixed concentration of PEI (100 µg mL^−1^), as depicted in Figure 3. The plate was covered with a transparent seal, the lid was added, and the covered plate was incubated for 1 h at 37 °C in darkness to allow the diffusion of CHL and PEI into the agar through the membrane. Afterward, the plate was exposed to the red light. For viable cell enumeration, the biofilm biomass was resuspended in 10 mL phosphate-buffered saline (PBS) (137 mM NaCl, 2.7 mM KCl, 10 mM Na_2_HPO_4_, 1.8 mM KH_2_PO_4_, pH 7.4) by vigorous vortexing and serially diluted.

### 4.6. Induction of Stringent Response

Induction of persister cells via amino acid starvation was performed using serine hydroximate (SHX, Sigma-Aldrich, St Louis, MO, USA) as previously described [34]. An overnight culture was diluted to an OD_600_ = 0.05 in M9 (1 mM MgSO_4_, 47 mM Na_2_HPO_4_·12H_2_O, 22 mM KH_2_PO_4_, 9 mM NaCl, 18 mM NH_4_Cl, 10 mM glucose) and grown under shaking at 37 °C for 2.5 h. Then, 500 µM SHX was added, and incubation was continued for 2 h under the previous condition; a culture without SHX was considered as a control, with inoculum size ~4 ± 1 × 10^7^ CFU mL^−1^. Subsequently, the cultures were directly exposed to photodynamic inactivation. Viable cell count was determined using the microdilution method. For confirmation of starvation induction, the expression levels of *relA*, *spoT* and *lon* were assessed via RT-PCR.

### 4.7. RNA Preparation and Quantitative RT-PCR

Two mL aliquots were taken from the previously prepared cultures for RNA extraction. Cell pellets were resuspended in 100 µL Tris-EDTA (TE) buffer with 1 mg mL^−1^ lysozyme and incubated at 37 °C for 10 min. Total RNA was isolated using the NucleoSpin RNA kit protocol 5.2 (Macherey-Nagel, Düren, Germany) according to the instructions of the manufacturer. RNA quality and quantity were assessed using a NanoDrop spectrophotometer (Thermo Fisher Scientific, Wilmington, DE, USA), and all RNA samples were found to have a 260/280 nm absorbance ratio of 2.17 ± 0.02. RNA samples were stored at −80 °C. A total of 700 ng of the single RNA preparations were converted to cDNA using the Quantitect reverse transcription kit (Qiagen, Hilden, Germany) following the instructions of the manufacturer.

Primers for PCR amplification of cDNA are listed in Table 1. A Bio-Rad (Munich, Germany) thermal cycler c1000 Touch CFX96-Real-Time system was used for the quantification of the cDNA. Triplicate PCR reactions were performed using the Luna universal qPCR master mix (New England Biolabs, Frankfurt am Main, Germany). Eight microliters of 10-fold dilution of cDNA and 0.5 µL of primers (10 μM), with a final concentration of 250 nM used in the total volume of 20 µL. qPCR cycling conditions were adjusted to 95 °C, 1 min for the initial denaturation, followed by 40 cycles of 95 °C, 15 s, 60 °C, 30 s, 72 °C and 30 s with data acquisition. A melt curve was run at the end of the 40 cycles using a temperature range from 60 to 95 °C to confirm the presence of a unique PCR reaction product. To check for residual contaminating genomic DNA, negative controls without reverse transcriptase were analyzed in the same way. The amount of signal in the controls was usually close to the non-template control. Reaction efficiencies were calculated for each amplicon. Quantitative cycle (Cq) values were generated using the Bio-Rad CFX Maestro software 2.3 (Biorad CFX, Germany) and used to quantify the stability of the reference gene using the 2^−ΔCt^ method [59]. The Cq values were also used to quantify the relative gene expression to the reference gene *rpoS* by the 2^−ΔΔCt^ method [59].

### 4.8. Statistical Analysis

The influence of growth medium and incubation time on biofilm formation was analyzed via one-way ANOVA, the TUKEY test (*p* < 0.05) [62]. The matrices of the dose–response and ZIP synergy score were plotted using the RStudio SynergyFinder R package (version 2023.03.0+386) [63]. The synergy score range from 0 to 100, with a higher value indicating better efficacy and a ZIP score lower than 0 indicating antagonism [63]. In this study, the zero interaction potency (ZIP) was utilized as a reference model to examine the interaction between CHL and PEI because the ZIP method calculates a synergy score based on a mathematical model considering the dose–response relationship of each antimicrobial agent individually and in combination. In addition, it assumes that antimicrobial agents are independent and do not interact with each other when combined, allowing significant and precise definitions of synergy, additivity and antagonism in the study of antimicrobial interaction [64].

## 5. Conclusions

It is well discussed in the literature that anionic PS has no lethal effect on Gram-negative bacteria [24,28]. However, our study demonstrates that high concentration and more exposure time of CHL to the red light is lethal to *P. aeruginosa* planktonic cells and combination with PEI potentiates lower concentrations of CHL to exert the same lethal effect. In addition, the synergism between CHL and PEI is obvious against the biofilm and persister cells. Overall, this study gives new insights into the aPDI of CHL against *P. aeruginosa.*

## Figures and Tables

**Table 1:** Primers used in this study.

Gene	Sequence (5′->3′)	Primer Size (b)	Product Size (bp)	Reference
*relA*	GAGATCCCATCGTCGGCTAC	20	171	This study
	CATAGGCACGGATCGCGATA	20		
*spoT*	CGACAAGGTCGATACCTGCT	20	195	This study
	TTGGCCATCTCTTCCATCTC	20		
*lon*	CCGTGGTGCGTTCCTACATA	20	138	This study
	GAATGCGCTCCTTGACCTCT	20		
*rpoS*	CTCCCCGGGCAACTCCAAAAG	21	200	[60,61]
	CGATCATCCGCTTCCGACCAG	21		

## Data Availability

The data presented in this study are available upon request from the corresponding authors.

## Supplementary Materials

The supporting information can be downloaded at: https://www.mdpi.com/article/10.3390/ijms241512098/s1.
