# Supplementary material for: Photodynamic Activity of Chlorophyllin and Polyethylenimine on Pseudomonas aeruginosa Planktonic, Biofilm and Persister Cells
Source: Int J Mol Sci. 2023 Jul 28;24(15):12098. doi: 10.3390/ijms241512098 (PMC10419130; doi:10.3390/ijms241512098)
Supplement: Supplementary file 1 [file ijms-24-12098-s001.zip › ijms-2497748-supplementary.pdf]

Supplementary material:

0

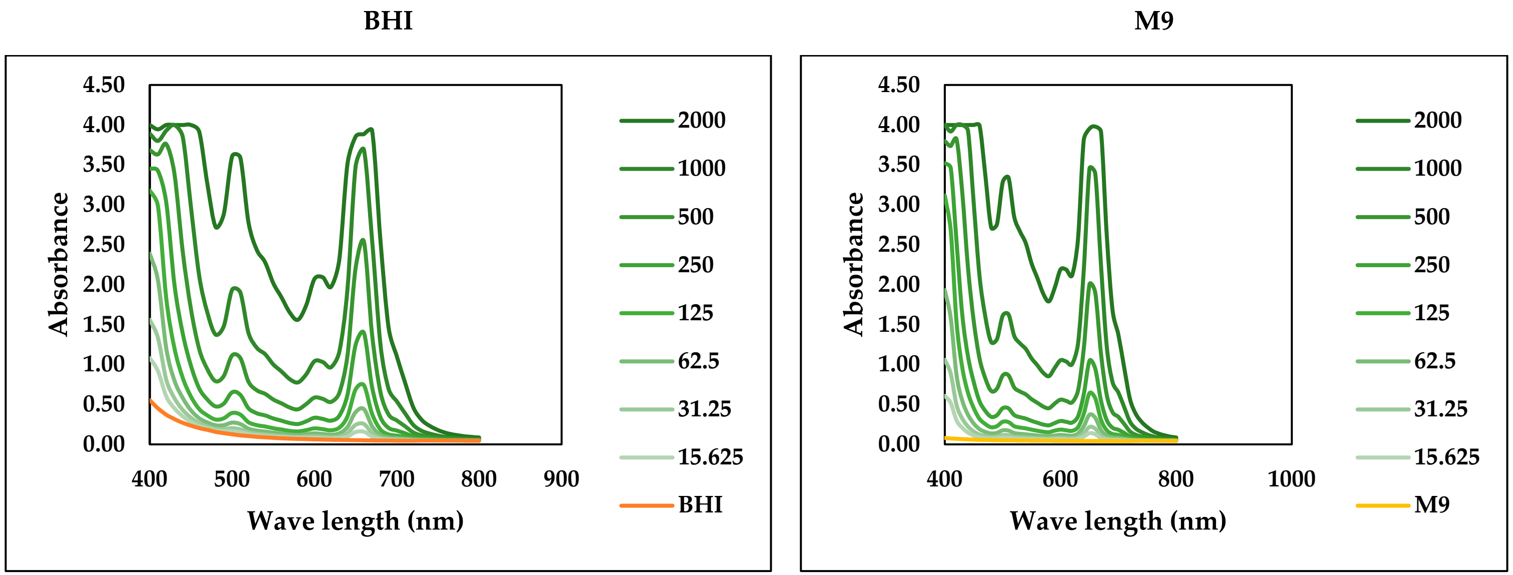

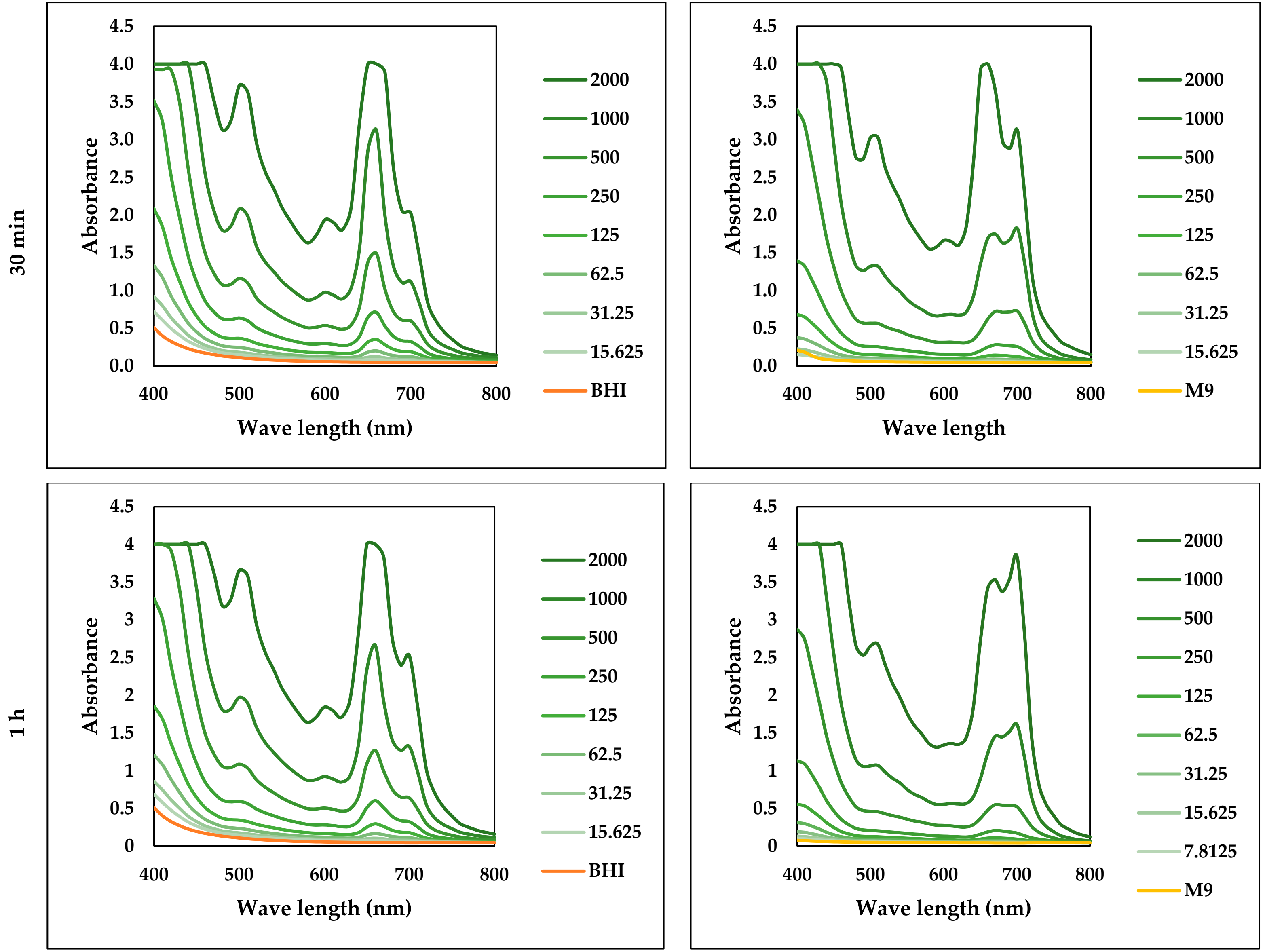
30 min
1 h
Absorbance
Wave length (nm)
Wave length
4.5
4.0
3.5
3.0
2.5
2.0
1.5
1.0
0.5
0.0
4
3
2
1
0
400
500
600
700
800
2000
1000
500
250
125
62.5
31.25
15.625
7.8125
BHI
M9

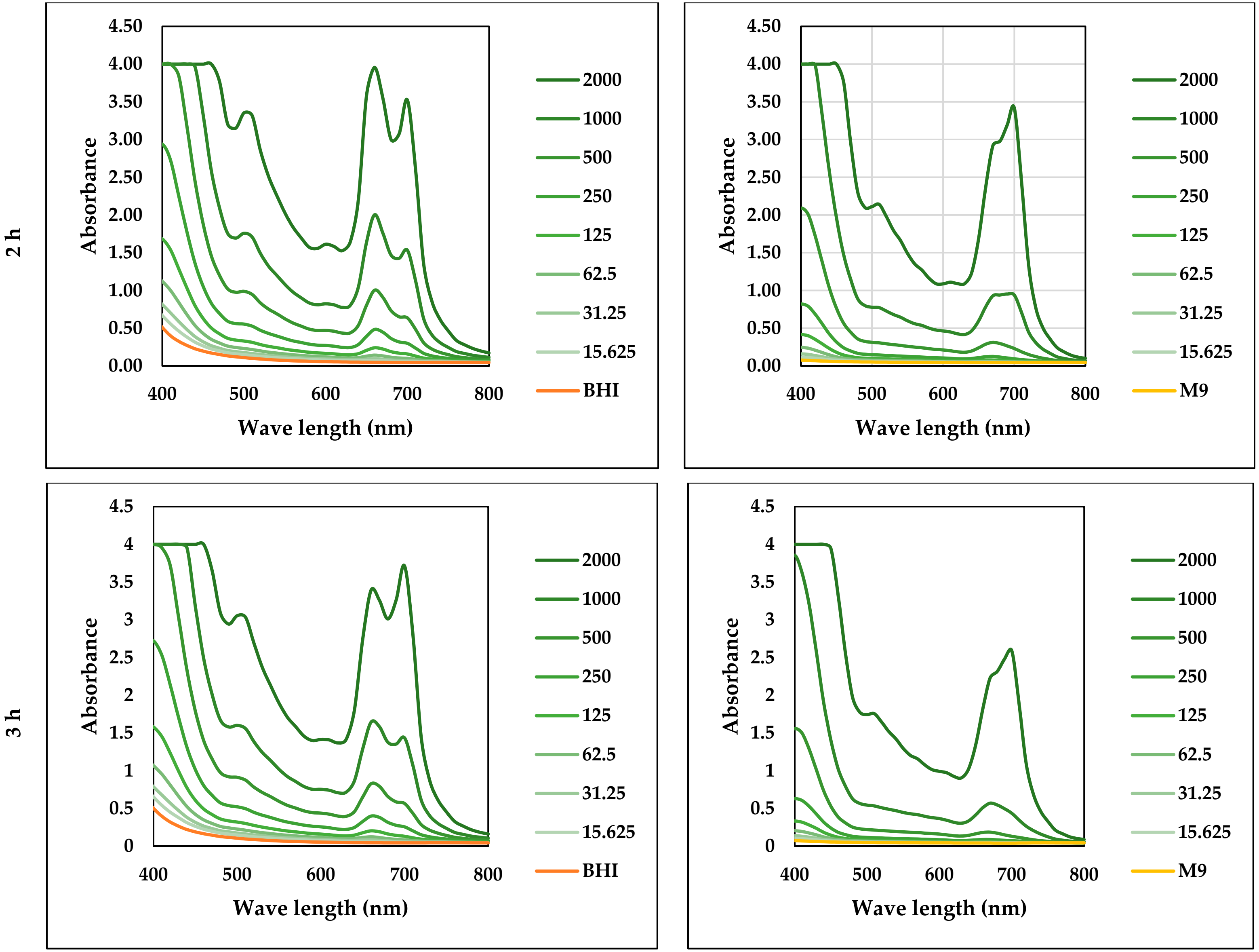
2 h
3 h
Absorbance
Wave length (nm)
4.50
4.00
3.50
3.00
2.50
2.00
1.50
1.00
0.50
0.00
400
500
600
700
800
2000
1000
500
250
125
62.5
31.25
15.625
BHI
M9
4.5
4
3.5
3
2.5
2
1.5
1
0.5
0

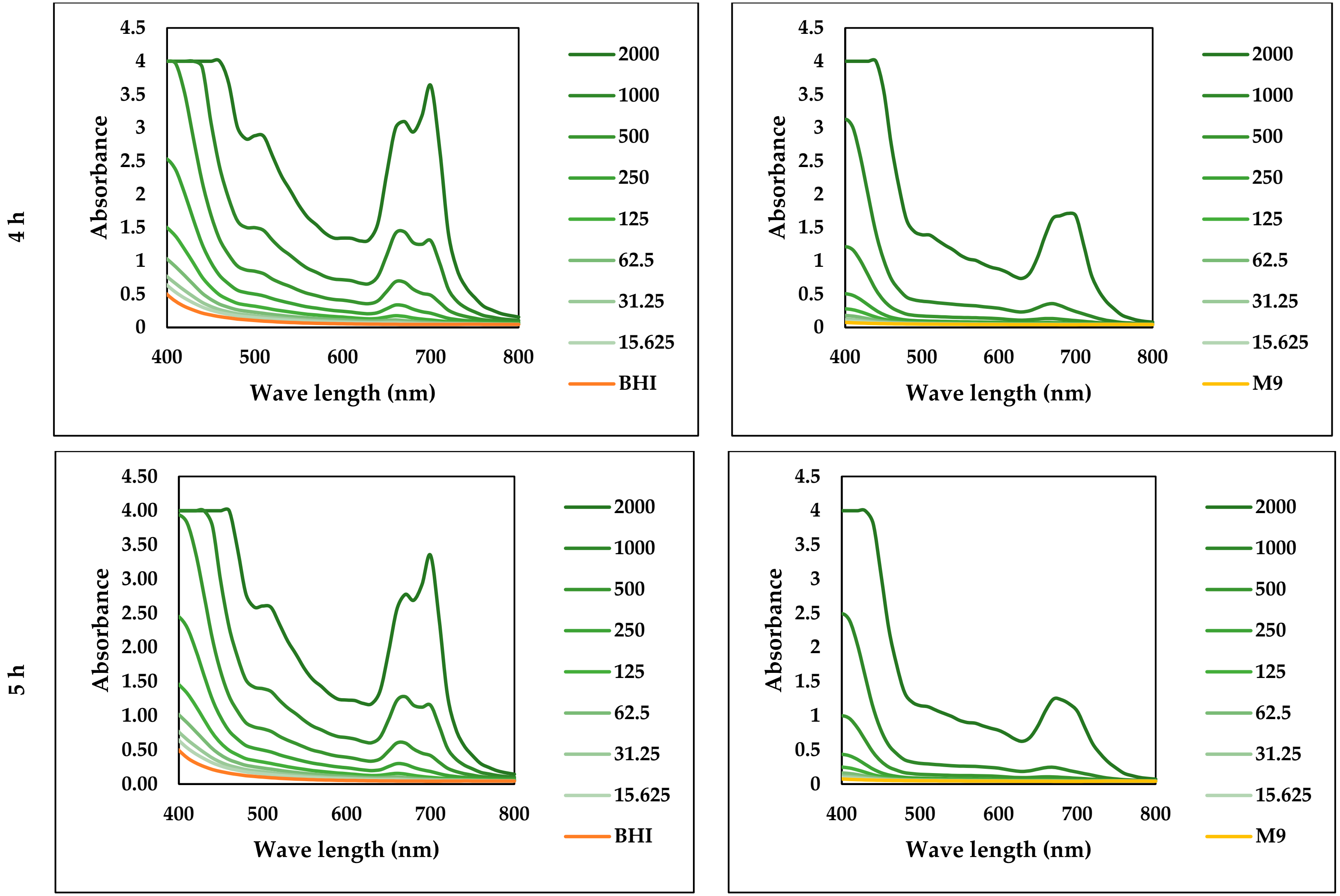
4 h
5 h
Absorbance
Wave length (nm)
4.5
4
3.5
3
2.5
2
1.5
1
0.5
0
400
500
600
700
800
4.50
4.00
3.50
3.00
2.50
2.00
1.50
1.00
0.50
0.00
2000
1000
500
250
125
62.5
31.25
15.625
BHI
M9

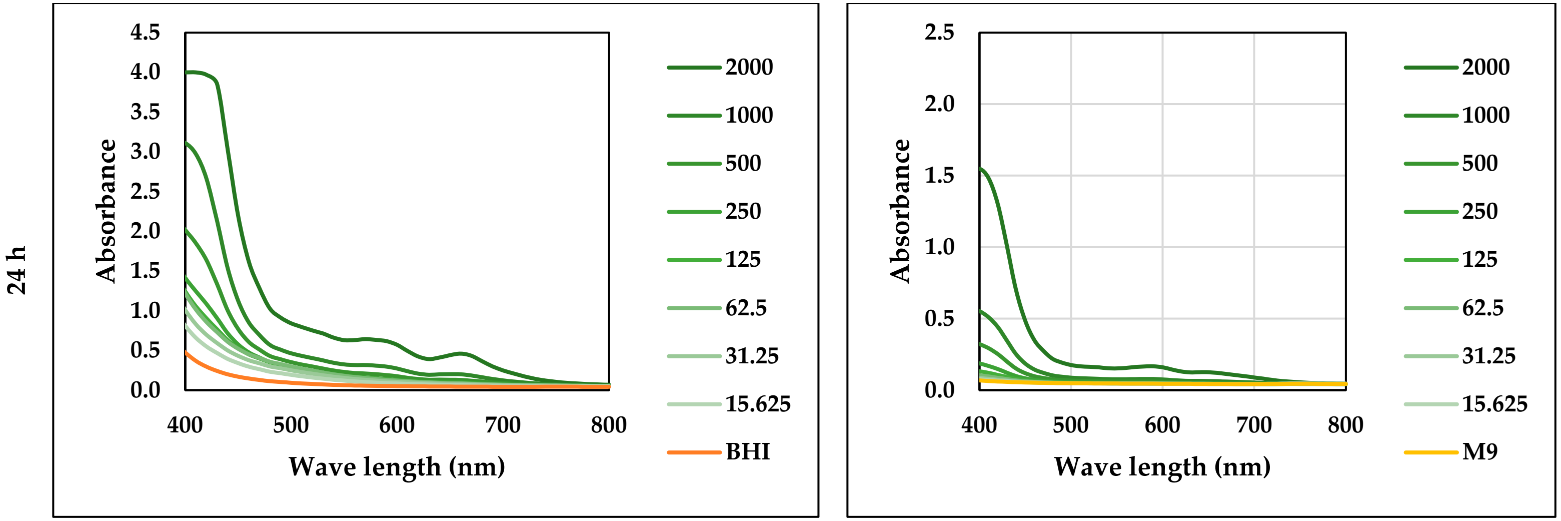

Figure S1: Absorption spectrum of chlorophyllin in BHI and M9 during irradiation over the designated time points.

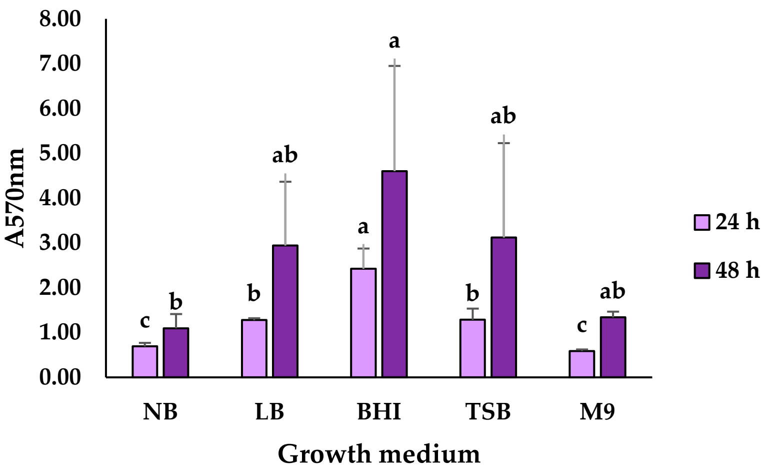

Figure S2: Influence of growth medium and incubation time on biofilm formation. The result is an average of four biological replicates. Error bars represents standard deviation of the mean.

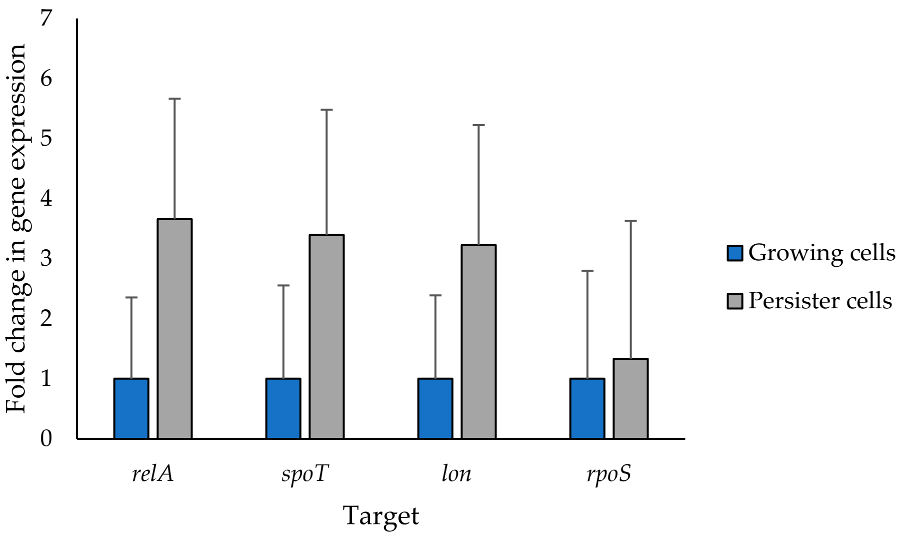

Figure S3: The relative fold change in gene expression of *P. aeruginosa* cells following treatment with SHX compared to control untreated samples using the method $2^{-\Delta Cq}$.
